# An annotated checklist and species accounts of the native terrestrial reptiles of the Turks and Caicos Islands

**DOI:** 10.3897/zookeys.1290.198843

**Published:** 2026-08-17

**Authors:** R. Graham Reynolds

**Affiliations:** 1 Department of Biology, University of North Carolina Asheville, 1 University Heights, Asheville, NC 28804, USA Department of Biology, University of North Carolina Asheville, 1 University Heights Asheville United States of America

**Keywords:** Bahamas, Caribbean, conservation, herpetofauna, inventory, Lucayan Archipelago

## Abstract

The Turks and Caicos Islands (TCI), an archipelago of more than 200 islands and islets at the southeastern terminus of the Lucayan Archipelago, support a native terrestrial reptilian fauna of 11 species representing eight families. Of these, eight species are endemic to the TCI, two non-endemic species are each represented by an endemic subspecies, and one species occurs in the TCI without endemic differentiation. Four subspecies are recognized within two of the endemic species. Seven species are of significant conservation concern, particularly the Critically Endangered *Spondylurus turksae* (likely now restricted to a single population) and the Endangered *Cyclura carinata*. Here, an annotated checklist is presented with illustrated species accounts for all native terrestrial reptiles of the TCI, synthesizing published literature through 2026 with original field data collected across the Turks and Caicos Banks between 2006 and 2025. Each account provides information on taxonomy, type specimen data, description, distribution within the archipelago, habitat use, natural history, and IUCN conservation status. Species accounts are accompanied by original photographs. I also recommend recognizing two subspecies of *Leiocephalus psammodromus*, instead of six (*L. p. psammodromus* for the Turks Bank and *L. p. apocrinus* for the Caicos Bank). This checklist provides a current reference for researchers, conservation practitioners, and natural historians working in the TCI and the broader Caribbean region.

## Introduction

**Figure 1.**
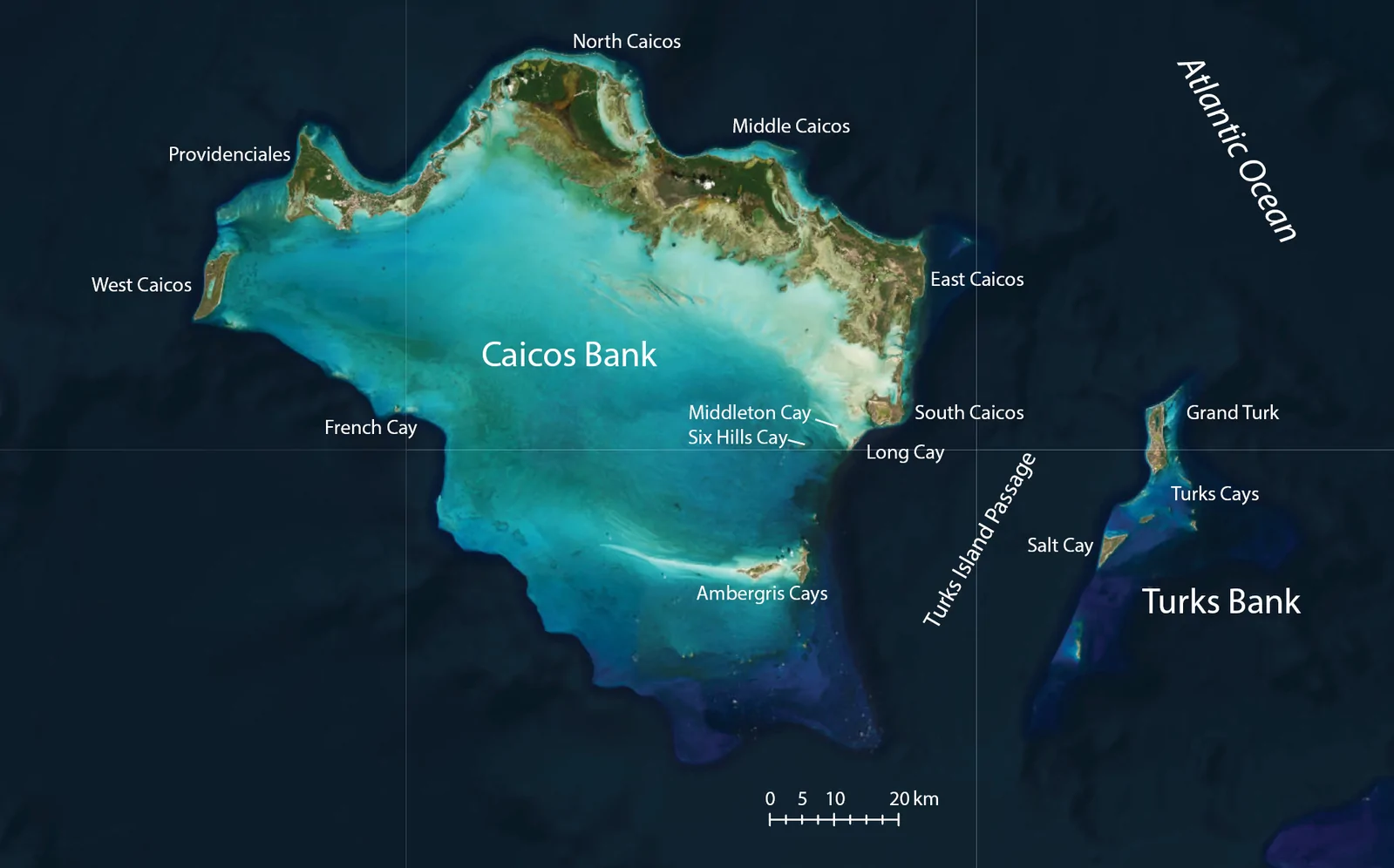
Map of the Turks and Caicos Islands, showing major islands mentioned in the text. Satellite base map generated in ArcGIS Pro.

The Turks and Caicos Islands (TCI), a British Overseas Territory, are located at the southeastern terminus of the Lucayan Archipelago, also called the Bahamian Archipelago, which includes the much larger Commonwealth of the Bahamas (approximately between latitudes 20°59'N and 21°58'N and longitudes 70°55'W and 72°30'W). The TCI consists of more than 200 islands and islets ranging in size from less than 1 ha to 144 km^2^ (Buckner et al. 2012), distributed across two shallow carbonate platforms: the Turks Bank to the east and the Caicos Bank to the west (Fig. 1). These banks are geologically distinct platforms of marine sediments overlain by oolitic limestone and separated from other banks in the archipelago (Sealey 2006). The Turks and Caicos banks are separated from one another by the narrow but deep (> 2,000 m) Turks Island Passage and have never been joined (Lighty et al. 1982; Fairbanks 1989). During the last glacial period, approximately 8,000–25,000 years before present, both banks were subaerial as large landmasses; rising sea levels since then have fragmented each bank into the present-day configuration of islands (see Kerans et al. 2019). The total land area of the TCI is approximately 500 km^2^, with the eight largest islands being Grand Turk, Salt Cay, Providenciales, North Caicos, Middle Caicos, East Caicos, West Caicos, and South Caicos (Fig. 1), with a maximum elevation of approximately 50 m above sea level.

The TCI lie approximately 130 km north of Hispaniola, and the terrestrial herpetofauna of the region is largely derived from Hispaniola, following ocean currents and hurricane tracks that likely facilitated over-water dispersal (Schwartz 1967, 1968; Hedges 2006; Reynolds 2011). Vegetation across the archipelago consists of low coastal scrub on most islands, interspersed with coppice on larger islands and remnant tropical dry forest on North and Middle Caicos. Annual rainfall averages approximately 750 mm on the Caicos Bank and slightly less on the Turks Bank, with most precipitation occurring from September through December (Bosart and Schwartz 1979; Spellman et al. 2021). A northwest-to-southeast rainfall gradient exists, with the northwestern TCI receiving more rainfall than the drier southeastern portions (Sealey 2006; Reynolds 2011).

The terrestrial reptilian fauna of the TCI consists of 11 native species. Of these, eight species are endemic to the TCI, two non-endemic species are each represented by an endemic subspecies, and one species occurs in the TCI without endemic differentiation. Four subspecies are recognized within two of the endemic species. The native terrestrial reptilian fauna includes representatives of the families Iguanidae (rock iguanas), Leiocephalidae (curly-tailed lizards), Anolidae (anoles), Mabuyidae (skinks), Sphaerodactylidae (dwarf geckos), Typhlopidae (blindsnakes), Tropidophiidae (tropes), and Boidae (boas).

**Figure 2.**
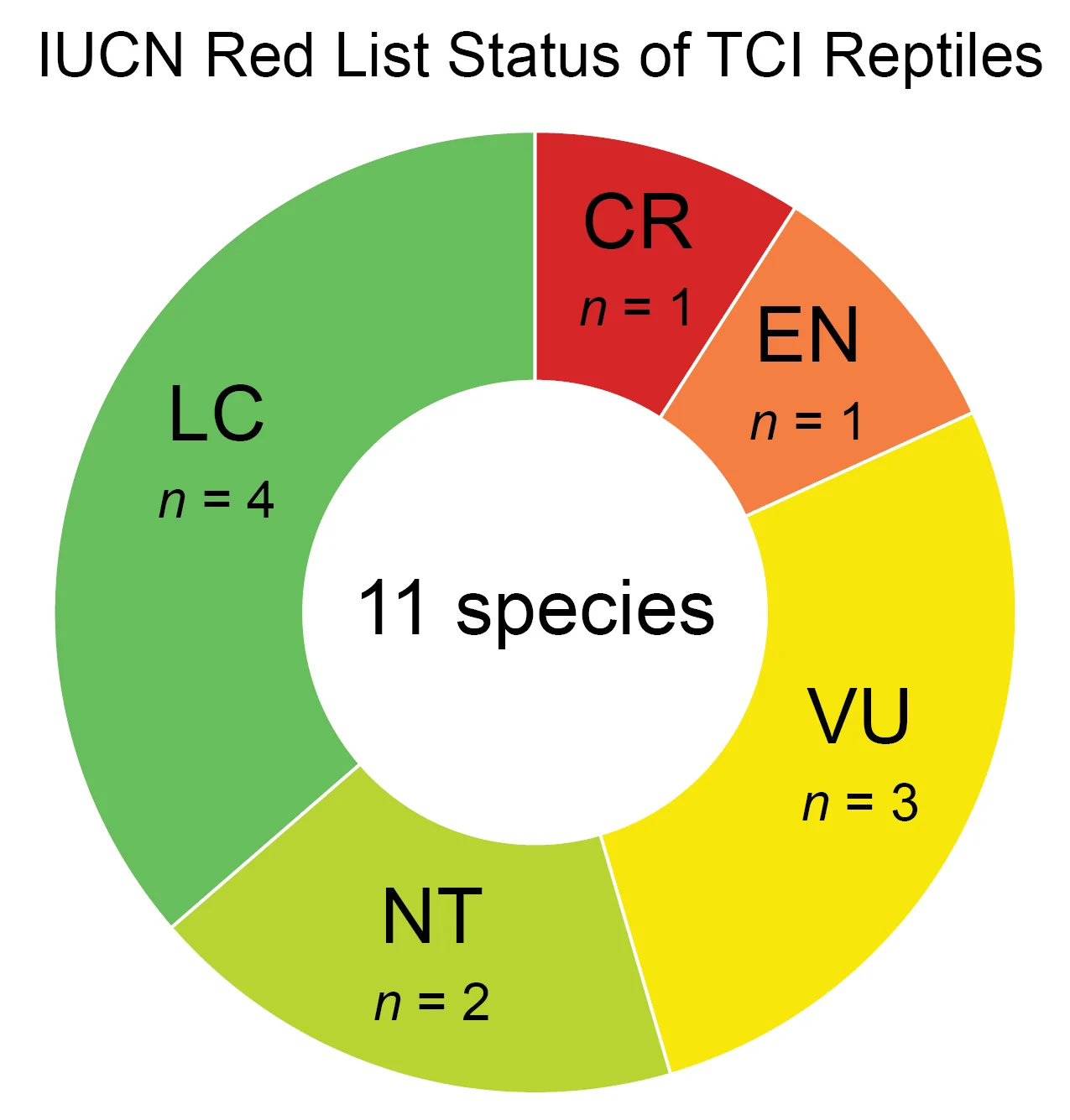
IUCN Red List conservation status of 11 native terrestrial reptile species in the Turks and Caicos Islands. Abbreviations: CR, Critically Endangered; EN, Endangered; VU, Vulnerable; NT, Near Threatened; LC, Least Concern. Data from IUCN Red List of Threatened Species (Version 2024-2).

One species is considered Critically Endangered on the International Union for the Conservation of Nature (IUCN) Red List, one species is Endangered, three are Vulnerable, and two are considered Near Threatened (Fig. 2). Over a dozen introduced reptilian species have been documented in recent decades, including known introductions through the ornamental plant trade, the pet trade, and importation of goods from Florida, USA, although the impacts of these on native species are unknown (Reynolds and Niemiller 2010; Buckner et al. 2012; Burgess 2012; Colosimo et al. 2020).

This annotated checklist and accompanying species accounts compile published information on the native terrestrial reptiles of the TCI through 2026. Each species account provides information on taxonomy, description, distribution within the TCI, habitat use, natural history, and conservation status, building primarily from Reynolds (2011) and Buckner et al. (2012) while adding summaries of dozens of studies conducted and/or published since 2006; and species accounts are accompanied by one or more in-situ photographs illustrating each species (by the author, unless otherwise indicated). Some natural history observations by the author are listed here as (pers. obs.) if they are novel to this manuscript or are in preparation for publication elsewhere and are based on fieldwork by the author annually between 2006–2025.

## Species accounts

The following abbreviations are used throughout this manuscript: **AMNH** (American Museum of Natural History); **ANSP** (Academy of Natural Sciences of Drexel University); **APSU** (Austin Peay State University); **CM** (Carnegie Museum of Natural History); KU (University of Kansas Natural History Museum); **MCZ** (Museum of Comparative Zoology); **SBH** (S. Blair Hedges); **SVL** (snout-vent length); **TCI** (Turks and Caicos Islands); **TL** (tail length); **TOL** (total length); **USNM** (National Museum of Natural History, Smithsonian Institution).

Common names and taxonomy largely follow Hedges et al. (2019, 2026). Type specimens are provided when the types are from the TCI. For other species, accessioned photographic-voucher information is provided for individuals from the TCI. All photographs are by the author unless otherwise indicated.

### Family Iguanidae—Iguanas

#### Cyclura carinata

Taxon classificationAnimaliaSquamataIguanidae

Harlan, 1824

7052A8A4-7A97-5D75-BFF1-F68D9BB3D310

Fig. 3

##### Common name.

Turks & Caicos Iguana.

**Figure 3.**
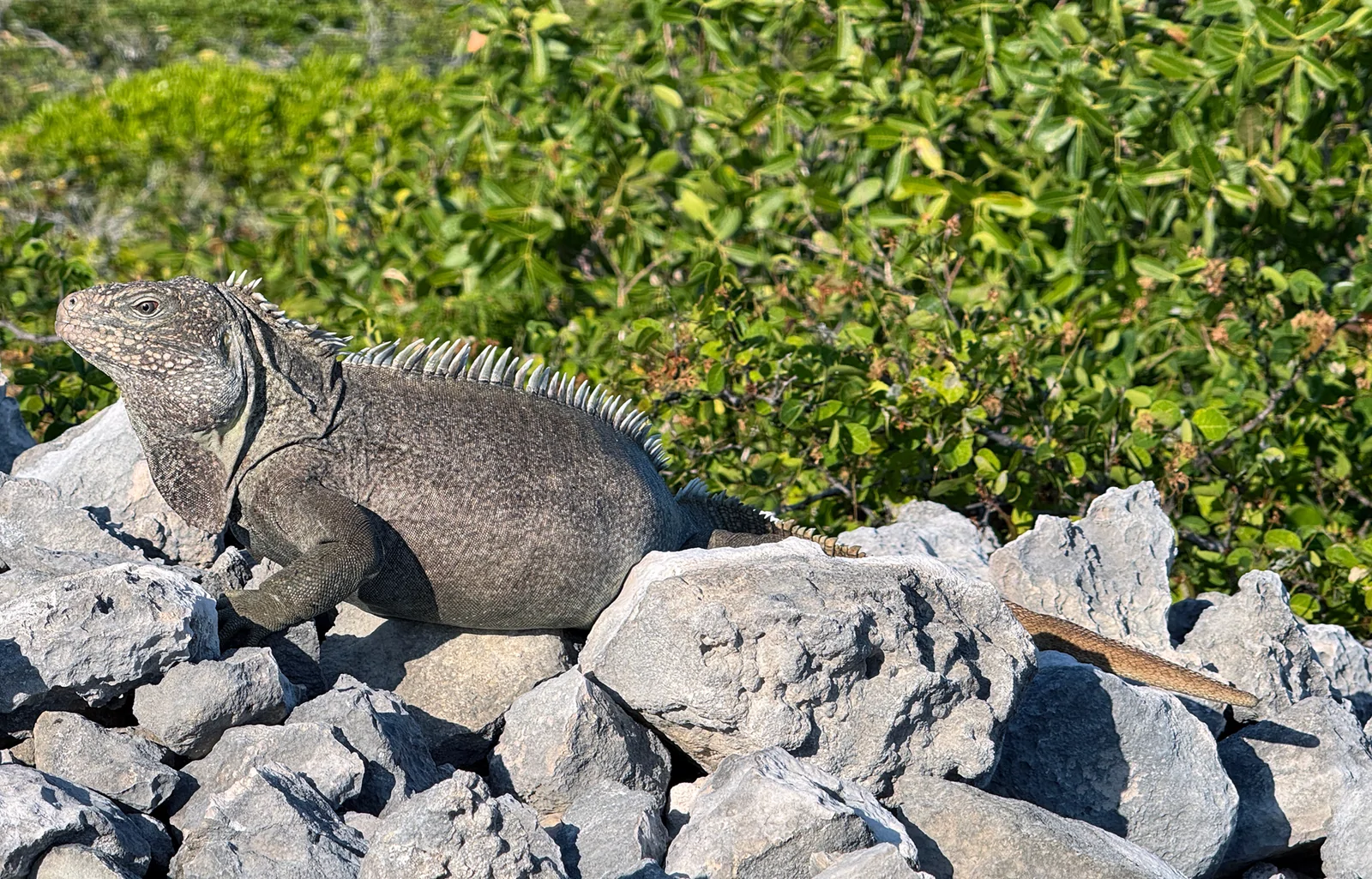
Adult male *Cyclura carinata* from Big Ambergris Cay, Caicos Bank.

##### Type material.

Holotype is unlocated, from “Turks Island”.

##### IUCN status.

This species has been assessed as Endangered (EN) on the IUCN Red List (Gerber et al. 2020).

##### Taxonomy.

*Cyclura carinata* is a member of the Hispaniolan rock iguana diaspora and is a sister species to *C. ricordii* (Reynolds et al. 2022).

##### Endemic status.

This species is endemic to the Turks and Caicos Islands; however, a single population occurs on Booby Cay, a 0.51-km^2^ islet off the eastern coast of Mayaguana, Bahamas. The Booby Cay population was formerly recognized as the subspecies *C. carinata bartschi*, but molecular analysis has shown that it is not sufficiently distinct to warrant subspecific status and was likely introduced from the Caicos Bank (Bryan et al. 2007; Welch et al. 2017).

##### Description.

*Cyclura carinata* is the smallest species among the nine recognized members of the genus *Cyclura* (Schwartz and Henderson 1991; Reynolds et al. 2022). Adults are robust and heavily built; adult males reach 400 mm SVL and weigh ~1.2–2.8 kg. Females are smaller, with an SVL of ~300 mm and mass between 0.5–1.5 kg (Schwartz and Henderson 1991; Colosimo et al. 2024). Males develop larger dorsal crests and femoral pores than females and have a more robust head and jaws. Dorsal coloration in adults is generally gray to brownish gray with variable banding, and juveniles tend to have cross-banding patterns on the dorsum.

##### Distribution in the TCI.

The species historically occurred on most islands in the TCI with suitable habitat but has been extirpated from 90% of its former range, including from most major islands in the archipelago, and now survives primarily on small cays that lack introduced vertebrate predators (Iverson 1978; Reynolds 2011; Gerber et al. 2020). The largest remaining populations occur on Big Ambergris Cay and Little Ambergris Cay on the southeastern edge of the Caicos Bank, where an estimated 15,000–20,000 individuals were counted prior to the start of large-scale development of Big Ambergris Cay (Gerber et al. 2020). Additional populations occur on small predator-free cays, including on some of the Caicos Cays and Turks Cays (Buckner et al. 2012). The population on Long Cay (Caicos Bank) was reintroduced following cat eradication (Mitchell et al. 2002), and populations have been translocated to numerous other small islands throughout the archipelago (Gerber 2007; Havery et al. 2021).

##### Habitat.

*Cyclura carinata* occupies xeric to semi-arid habitats on the limestone and sandy cays and islands of the TCI. Preferred habitats include low coastal scrub, cactus-dominated xeric shrubland, and sandy areas with substrate suitable for burrowing (Iverson 1979; Henderson and Powell 2009). Individuals use burrows or large rocks for overnight shelter and thermoregulation, whereas sand burrows are excavated for nesting. On islands where they occur, iguanas are commonly observed in open habitats with access to sun and shade, including beach edges, road margins, and open scrub (Iverson 1979).

##### Natural history.

Turks and Caicos Rock Iguanas are primarily herbivorous, feeding on a wide variety of plant material, including leaves, flowers, and fruits, and occasionally consuming invertebrates, especially by younger individuals (Iverson 1979; Auffenberg 1982). Nesting occurs from May through July, and clutch sizes are relatively small, with females laying 2–6 eggs that incubate ~85–100 days (Gerber and Iverson 2000).

##### Conservation.

*Cyclura carinata* is one of the most intensively studied and managed of all West Indian iguanas (Lemm and Alberts 2011). Conservation efforts began with Iverson (1978, 1979) on Pine Cay (Caicos Cays), and long-term monitoring and translocation programs have been ongoing (Alberts 2001; Alberts and Gerber 2004; Gerber 2007). Cat-eradication on Long Cay (Caicos Bank) in 1999 led to the successful reintroduction of iguanas from Big Ambergris Cay (Mitchell et al. 2002). This population has since established successfully, with juvenile recruitment observed (pers. obs.).

*Cyclura carinata* is susceptible to predation by introduced mammals, particularly feral cats and dogs. Iverson (1978) documented the near extirpation of iguanas from Pine Cay (Caicos Cays) within a few years following the establishment of a feral cat population. Cats preferentially prey on juveniles and can effectively prevent recruitment. Semi-feral goats and donkeys also degrade habitat quality by eliminating ground cover, food plants, and nesting substrate.

### Family Leiocephalidae—Curly-tailed lizards

#### Leiocephalus psammodromus

Taxon classificationAnimaliaSquamataLeiocephalidae

Barbour, 1916

25A8C290-4132-594C-9CC1-34CB2F44AB3E

##### Common name.

Turks and Caicos Curlytail.

#### Leiocephalus psammodromus psammodromus

Taxon classificationAnimaliaSquamataLeiocephalidae

Barbour, 1916

32D593FE-6681-52B4-82E8-E992948BABAD

Fig. 4A

##### Type material.

Holotype (*L. p. psammodromus*) MCZ 11948, Big Sand Cay, Turks Bank.

**Figure 4.**
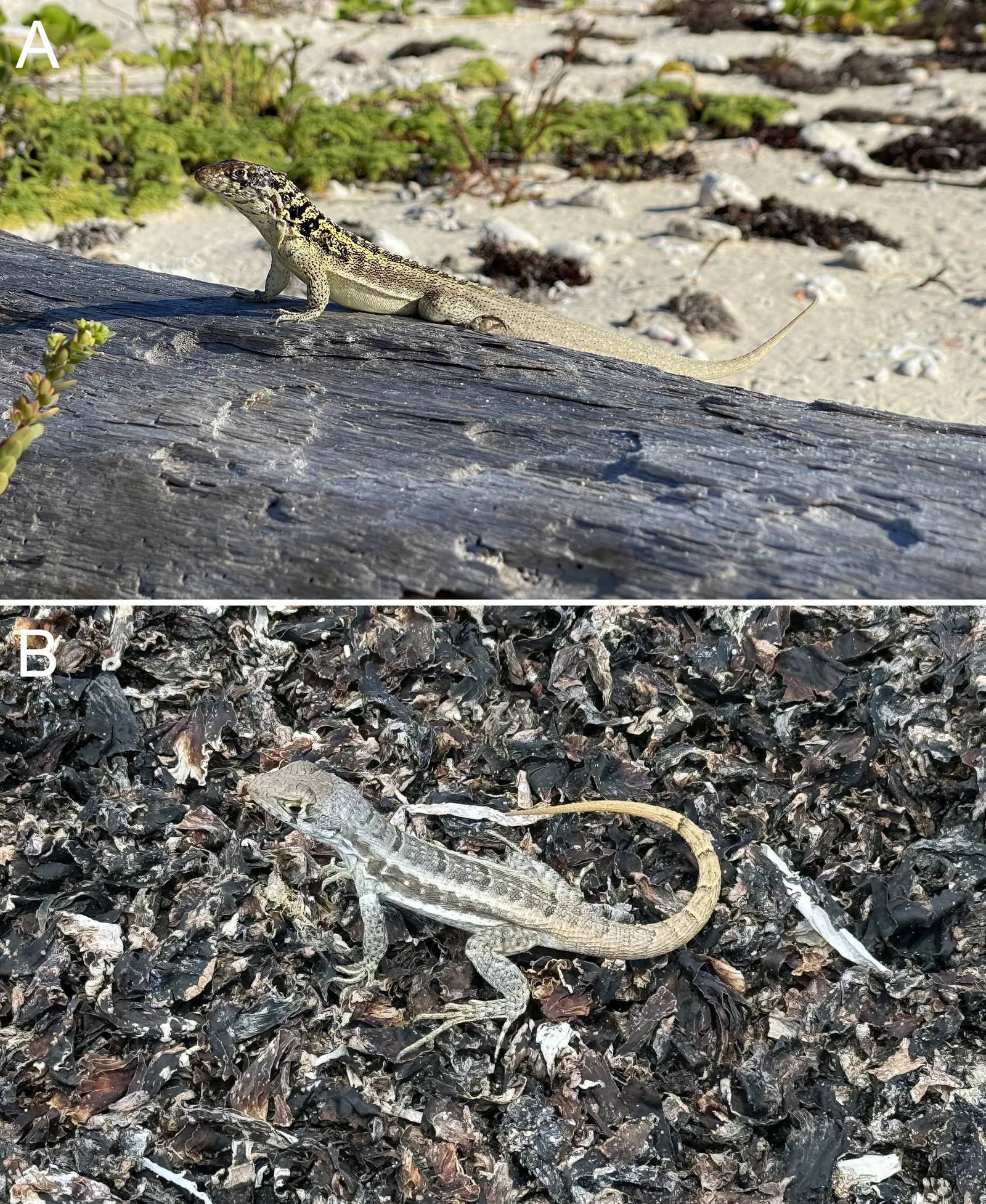
**A**. Adult male *Leiocephalus p. psammodromus* from East Cay, Turks Bank; **B**. Adult female *Leiocephalus p. apocrinus* from Big Ambergris Cay, Caicos Bank.

#### Leiocephalus psammodromus apocrinus

Taxon classificationAnimaliaSquamataLeiocephalidae

Schwartz, 1967

89851422-AFC1-5442-885B-10BCF88047F1

Fig. 4B

##### Type material.

Holotype (*L. p. apocrinus*) CM 40601, Ambergris Cays, Caicos Bank.

##### IUCN status.

This species has been assessed as Vulnerable (VU) on the IUCN Red List (Reynolds 2016a).

##### Taxonomy.

Six subspecies have been described (Schwartz 1967; Schwartz and Henderson 1991): *L. p. aphretor* (Grand Turk and Turks Cays), *L. p. apocrinus* (Big and Little Ambergris Cays), *L. p. arenarius* (Big Sand Cay), *L. p. cacodoxus* (Providenciales), *L. p. hyphantus* (Caicos Cays), and *L. p. mounax* (South Caicos and Long Cay). However, molecular genetic analysis of 259 individuals from 13 islands showed two reciprocally monophyletic lineages (one on each bank) and possibly a third, cryptic lineage present on both banks, a pattern that does not correspond to current subspecific taxonomy and has not yet been supported by additional data or sampling (Reynolds et al. 2012). These lineages also do not correspond to the morphologically described subspecies, leading Reynolds et al. (2012) to recommend discontinued use of most subspecies. Because *L. p. apocrinus*, *L. p. cacodoxus*, *L. p. hyphantus*, and *L. p. mounax* were published simultaneously by Schwartz (1967) and no previous author has acted as First Reviser, I here act as First Reviser (ICZN Art. 24.2.2) and give precedence to *L. p. apocrinus* (*L. p. apocrinus* Schwartz, 1967 = *L. p. cacodoxus* Schwartz, 1967, syn. nov.; = *L. p. hyphantus* Schwartz, 1967, syn. nov.; = *L. p. mounax* Schwartz, 1967, syn. nov.) for the Caicos Bank populations, on the grounds that it was the first subspecies described by Schwartz (1967: 163) from the Caicos Bank. *Leiocephalus p. psammodromus* Barbour, 1916 has priority for the Turks Bank populations by date of publication and requires no First Reviser action.

##### Endemic status.

Endemic to the Turks and Caicos Islands.

##### Description.

*Leiocephalus psammodromus* is a medium-sized lizard that can curl its tail upward over its back, particularly when alert or disturbed. Adults are 80–110 mm SVL in males, and the species exhibits sexual dimorphism in coloration and body size, with males larger than females (50–60 mm SVL) and exhibiting a more colorful dorsal pattern (Schwartz and Henderson 1991; Smith 1992; Reynolds et al. 2012). Dorsal coloration is typically grayish to brownish, with variable patterns of spots or stripes depending on population; mature males often have yellows, blacks, and pinks on the dorsal and ventral surfaces (Fig. 4). The throat and gular coloration and color patterns vary by population, although no diagnostic characters distinguish the subspecies other than distribution and molecular data (Schwartz 1967; Reynolds et al. 2012).

##### Distribution in the TCI.

*Leiocephalus psammodromus* occurs on most major islands larger than ca 0.42 km^2^ on both the Turks and Caicos Banks (Reynolds 2011; Reynolds et al. 2012). The species occurs on most major islands of the Caicos Bank, including North Caicos, Middle Caicos, Providenciales, East Caicos, Big and Little Ambergris Cays, and Long Cay, as well as on the Caicos Cays (Pine Cay, Little Water Cay, Water Cay, and Fort George Cay). On the Turks Bank, populations persist on Gibbs Cay, Long Cay, and the Big Sand Cay group, and were recorded on East Cay during surveys in 2022–2023 (Reynolds 2023; Reynolds et al. 2024a). Some smaller cays on the Caicos Bank (e.g., Mangrove Cay, Middleton Cay) also lack this species (Reynolds et al. 2012). Reynolds et al. (2012) surveyed 19 islands across the TCI, finding the species on 13 and failing to detect it on six (four of which were considered extirpations). The species is considered extirpated from Grand Turk, Salt Cay, South Caicos, and Cotton Cay (Reynolds et al. 2012).

##### Habitat.

*Leiocephalus psammodromus* occupies a variety of xeric scrub habitats (Smith 1994, 1995). The species is common in beach scrub, open coppice, rocky coastal areas, open sandy areas, road margins, and around human habitation and gardens. It is tolerant of habitat disturbance, although it does not tolerate islands with well-established populations of cats or other predatory mammals (Reynolds et al. 2012). On East Cay (Turks Bank), *L. p. psammodromus* are found in grassy areas along beaches where sandy substrate is available for nesting and where abundant orthopteran prey, especially *Schistocerca serialis* grasshoppers, are concentrated (Reynolds 2023).

##### Natural history.

*Leiocephalus psammodromus* is a diurnal omnivore with a catholic diet, consuming a variety of arthropods, plant material (especially flowers), and occasionally smaller lizards (Smith 1994; Henderson and Powell 2009). Females produce small clutches of 1–4 eggs, with multiple clutches possible per season (Smith and Iverson 1993). Like other members of the genus, tail-curling behavior is used in social signaling, and the tail also can serve as a distraction to predators. *Leiocephalus psammodromus* is susceptible to predation by feral mammals, particularly cats (Iverson 1978; Smith and Iverson 1993).

##### Conservation.

Although widespread in the TCI, this species likely faces some local threats from feral mammal predation and habitat loss, and several populations appear to have been extirpated (South Caicos and Grand Turk; Reynolds et al. 2012; Reynolds 2016a).

### Family Anolidae—Anoles

#### Anolis scriptus scriptus

Taxon classificationAnimaliaSquamataAnolidae

Garman, 1887

AF597B08-18B9-5C42-A738-CFD25F0C095F

Fig. 5

##### Common name.

Turks and Caicos Anole.

**Figure 5.**
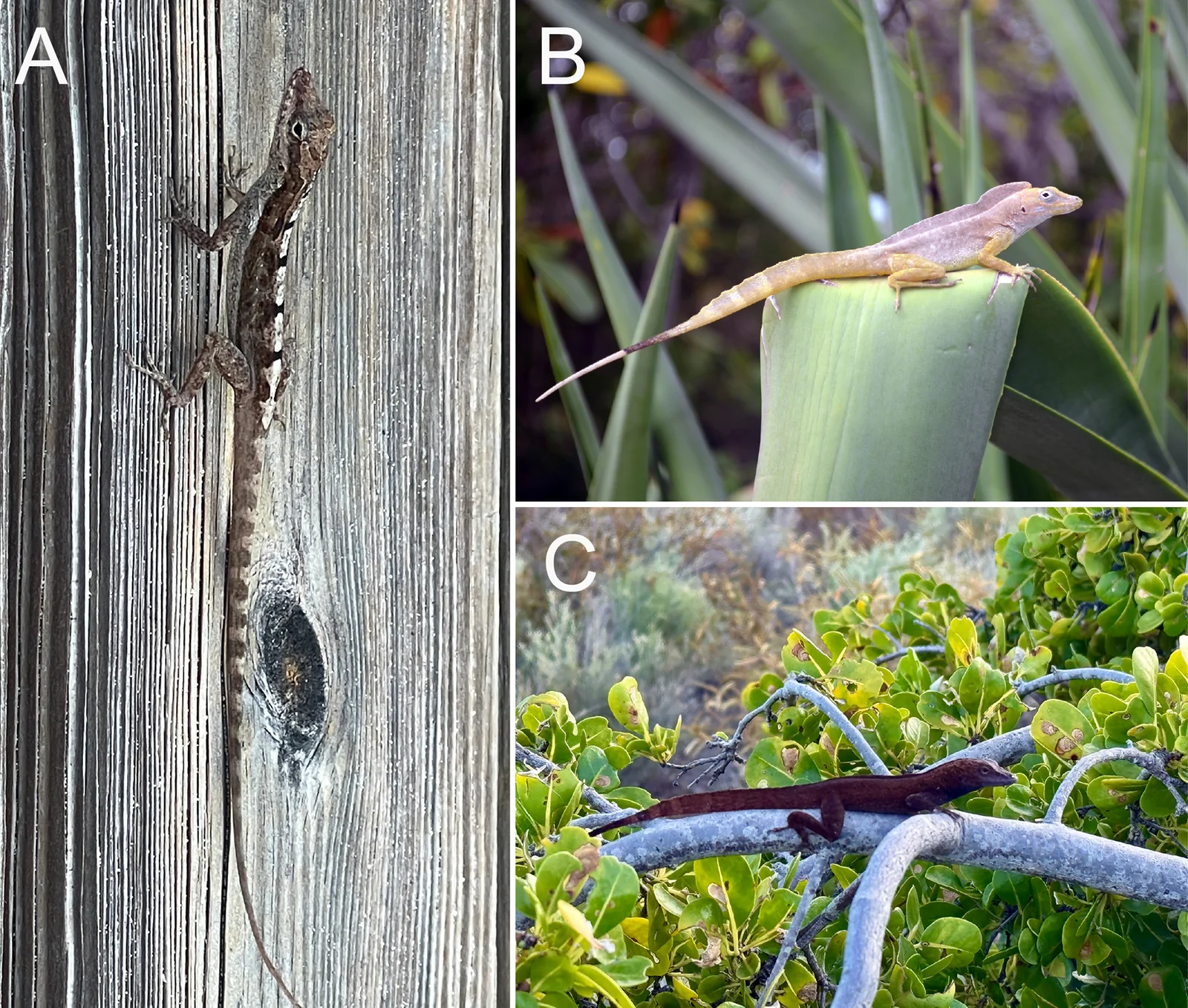
**A**. Adult female *Anolis s. scriptus* from Long Cay, Turks Bank; **B, C**. Two adult male *A. s. scriptus* from Big Ambergris Cay, Caicos Bank, showing the range of dorsal colors in the species, which can vary throughout the day.

##### Type material.

Lectotype MCZ 65950, “Silver Key” Turks and Caicos Islands (unknown locality).

##### IUCN status.

This species has been assessed as Least Concern (LC) on the IUCN Red List (Reynolds et al. 2020a).

##### Taxonomy.

The TCI population represents the subspecies *Anolis scriptus scriptus*, considered a subspecies of the Southern Bahamas anole complex (Rand 1962).

##### Endemic status.

The subspecies is endemic to the TCI.

##### Description.

*Anolis scriptus* is a small, slender lizard with well-developed adhesive toepads, like other members of the genus. Adults typically reach 40–65 mm SVL, with males slightly larger than females (Rand 1962). Dorsal coloration is variable, ranging from green to gray or brown with small vermiculation sometimes visible, and individuals can change color in response to temperature, stress, and social context, becoming very dark (particularly males; Fig. 5). The male dewlap color in TCI populations is typically yellow-orange to orange with variable dark pigment. Females lack a prominent dewlap and have much more patterning on the dorsum, ranging from a single midline white stripe to cross-banding and/or vermiculation (Fig. 5).

##### Distribution in the TCI.

*Anolis scriptus scriptus* is the most widely distributed native lizard in the TCI, occurring on all major islands and most small cays and rocky outcrops with some vegetation on both the Turks and Caicos Banks (Reynolds 2011; Buckner et al. 2012). The species has been recorded from North Caicos, Middle Caicos, East Caicos, West Caicos, Providenciales, Caicos Cays, South Caicos, Big and Little Ambergris Cays, Long Cay (Caicos Bank), Grand Turk, Salt Cay, and the Turks Cays (including Gibbs Cay, Long Cay, and East Cay) (Buckner et al. 2012; Reynolds 2023; Reynolds et al. 2024a).

##### Habitat.

*Anolis scriptus scriptus* is a habitat generalist, common in most vegetation types throughout the TCI, including xeric scrub, coppice, tropical dry forest, urban gardens, and areas with irrigated landscaping (Henderson and Powell 2009; Reynolds 2011). The species is edificarian, readily exploiting building surfaces, walls, and anthropogenic structures as perch sites. On remote cays, individuals tend to be more saxicolous (rock-dwelling), given the general absence of tall vegetation. For example, on East Cay Reynolds (2023) observed *A. scriptus* primarily on short vegetation on hillsides and among rocks, readily fleeing into crevices when approached.

##### Natural history.

*Anolis scriptus* feeds on small invertebrates from elevated perch sites on trunks, branches, walls, and rocks. Males are territorial and defend perches with dewlap displays, head-bobbing, and push-ups (Smith 1994). Females are less territorial but also engage in social signaling. Females lay single-egg clutches repeatedly throughout the warm season, burying eggs in moist soil. The species is consumed by multiple native predators in the TCI, including *Tropidophis greenwayi* and *Chilabothrus chrysogaster*, and is likely also eaten by *Leiocephalus psammodromus* (Smith 1994, 1995). Donihue et al. (2018) documented rapid, directional morphological selection in *A. s. scriptus* populations on Water Cay and Pine Cay (Caicos Cays) following Hurricanes Irma and Maria in 2017, finding that post-hurricane survivors had significantly larger toe pads than pre-hurricane individuals, a trait that proved heritable and not plastic (Donihue et al. 2020).

##### Conservation.

*Anolis s. scriptus* does not appear to be a conservation concern in the TCI (although nothing is known of the Bahamian populations of the species). The species is widespread, abundant, tolerant of habitat disturbance, and edificarian. It does not appear to have been extirpated from any island with sufficient habitat. Nevertheless, *Anolis sagrei*, a potential competitor and a potential predator (*Leiocephalus carinatus*; Lapiedra et al. 2018) have been introduced to Providenciales (Burgess 2012; Colosimo et al. 2020).

### Family Mabuyidae—Skinks

#### Spondylurus caicosae

Taxon classificationAnimaliaSquamataMabuyidae

Hedges & Conn, 2012

49FEDFD8-E19A-5422-9797-E49108023DF7

Fig. 6A

##### Common name.

Caicos Islands Skink.

**Figure 6.**
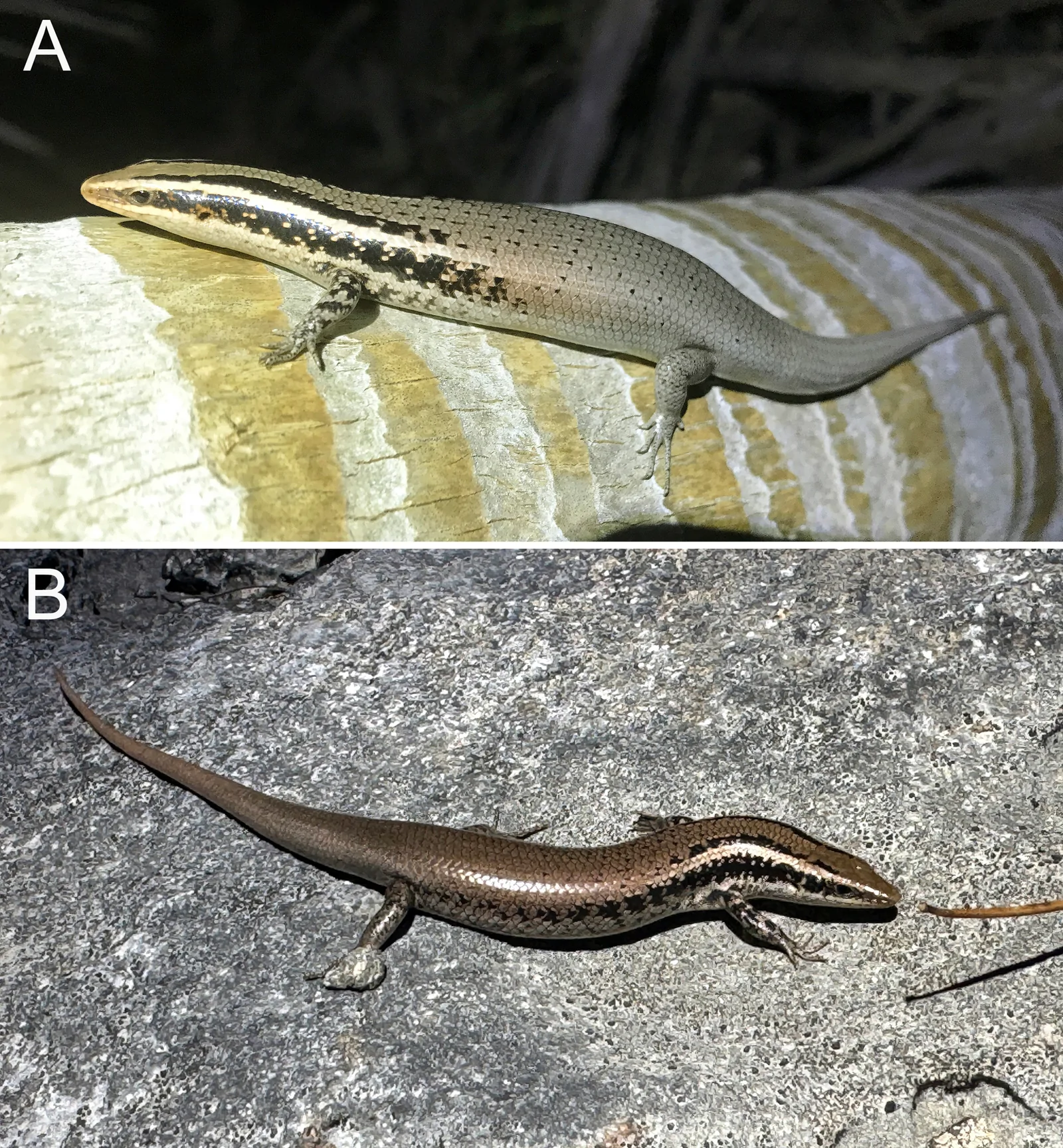
**A**. *Spondylurus caicosae* from Big Ambergris Cay, Caicos Bank. Note the relatively shorter length of the lateral stripe in comparison to *S. turksae*; **B**. *Spondylurus turksae* from Cotton Cay, Turks Bank.

##### Type material.

Holotype AMNH 80126, Long Cay (Caicos Bank).

##### IUCN status.

This species has been assessed as Near Threatened (NT) on the IUCN Red List (Reynolds and Hedges 2016a).

##### Taxonomy.

Formerly considered conspecific with the Virgin Islands Bronze Skink (*Spondylurus sloanii*; previously *Mabuya sloanii*), populations on the Caicos Bank were elevated to *Spondylurus caicosae* by Hedges and Conn (2012) based on morphological and molecular evidence. This species is visually distinguishable from the Turks Bank congener *S. turksae* by lateral black stripes extending only 1/3 of the way to the hindlimbs in *S. caicosae*, compared to stripes that continue (nearly) to the hindlimbs in *S. turksae*. Other differences include fewer total lamellae (196 vs. 211) and lower ear height (Hedges and Conn 2012), as well as significant phylogenetic divergence (Rivera et al. in press).

##### Endemic status.

Endemic to the Caicos Bank.

##### Description.

*Spondylurus caicosae* is a small, smooth-scaled lizard with a streamlined body and small limbs. Adults reach maximum body sizes of ~ 77.6 mm SVL and ~ 8.0 g (Hedges and Conn 2012). Dorsal coloration is generally brown with a coppery sheen, and lateral dark stripes extend posteriorly from the head to ~ 1/3 of the distance to the hindlimbs. The venter is pale or cream-colored. *Spondylurus caicosae* juveniles have a metallic silvery-colored tail (based on a single pers. obs. from Big Ambergris Cay), a previously undocumented trait.

##### Distribution in the TCI.

*Spondylurus caicosae* is distributed on the major islands of the Caicos Bank, including North Caicos, Middle Caicos, Providenciales, East Caicos, West Caicos, South Caicos, and some of the Caicos Cays and Big Ambergris Cay (Reynolds 2011; Buckner et al. 2012). Populations are likely present on most major islands and some smaller islands and cays, although this species is encountered extremely infrequently and might be extirpated in parts of its former range (Reynolds 2011). It is data-deficient regarding population status and distribution in the TCI.

##### Habitat.

*Spondylurus caicosae* can be found in xeric and (relatively) mesic upland habitats across the Caicos Bank, including in leaf litter, rock piles, and along forest edges. Individuals are typically encountered near rocks or in leaf litter, particularly in more shaded microhabitats within otherwise xeric environments on Big Ambergris Cay and North Caicos (pers. obs.).

##### Natural history.

No natural history studies have been published on the species. Based on the biology of related species in the genus *Spondylurus* and the broader family Mabuyidae, the species is presumed to be a diurnal forager feeding primarily on small arthropods (Henderson and Powell 2009). Mabuyid skinks are generally viviparous and this is expected for *S. caicosae*. Population ecology, reproductive biology, diet, and activity patterns in the TCI are unknown.

##### Conservation.

*Spondylurus caicosae*, like many Caribbean skinks, is probably in danger of being extirpated across portions of its range (Hedges and Conn 2012; Rivera et al. in press). It is likely highly susceptible to introduced predators (e.g., feral cats and rats). However, assessing conservation status is challenging given the limited knowledge of the species’ natural history, distribution, and responses to introduced species. Given the dire conservation status of its congener *S. turksae*, the species deserves conservation attention.

#### Spondylurus turksae

Taxon classificationAnimaliaSquamataMabuyidae

Hedges & Conn, 2012

0D7CDA81-E7D5-5FCB-ABFD-2A9D4EA8CBD2

Fig. 6B

##### Common name.

Turks Islands Skink.

##### Type material.

Holotype KU 242171, Grand Turk.

##### IUCN status.

This species is considered Critically Endangered (CR) on the IUCN Red List (Reynolds and Hedges 2016b) based on criteria B1ab and B2ab, triggered by a severe decline in the extent of occurrence and the area of occupancy (Reynolds and Hedges 2016b). Reynolds et al. (2024a) found that the species is likely now restricted to a single island (Cotton Cay).

##### Taxonomy.

Formerly considered conspecific with the Virgin Islands Bronze Skink (*Spondylurus sloanii*; previously *Mabuya sloanii*), populations on the Turks Bank were elevated to *Spondylurus turksae* by Hedges and Conn (2012) based on specimens from Grand Turk and Gibbs Cay. *Spondylurus turksae* is morphologically similar to *S. caicosae* of the Caicos Bank but differs in having lateral black stripes that continue to the hindlimbs (vs. extending only 1/3 of the way in *S. caicosae*), a higher number of total lamellae (211 vs. 196), and a higher ear height. Ongoing phylogenetic work indicates that *S. turksae* and *S. caicosae* are highly divergent from one another despite their morphological similarity (Hedges and Conn 2012; Reynolds et al. 2024a; Rivera et al. in press).

##### Endemic status.

Endemic to the Turks Bank.

##### Description.

*Spondylurus turksae* is a small, smooth-scaled lizard with a slender, streamlined body and relatively small limbs. Adults reach maximum known body sizes of ~ 79.1 mm SVL and 8.5 g, slightly larger than *S. caicosae* (Hedges and Conn 2012). The species has lateral black, semisolid stripes that extend along the flanks from the head to the hindlimbs. Dorsal coloration is generally brownish with a distinct coppery or metallic sheen.

##### Distribution in the TCI.

Historically, the species likely occurred across the Turks Bank, with documented records from Grand Turk, Gibbs Cay, and Cotton Cay, with presumed occurrence on Salt Cay and other Turks Bank islands (Reynolds 2011; Reynolds et al. 2024a). Barbour (1916) noted that skinks were rare on Grand Turk even then, suggesting population decline associated with the long history of human habitation and environmental modification on that island. SBH searched Grand Turk for skinks in August 1999 but found none (Hedges and Conn 2012).

Reynolds and Niemiller surveyed the Turks Cays in 2008 and discovered a new population on Cotton Cay (~113 ha), finding two individuals under rocks during diurnal surveys (Reynolds et al. 2024a). No skinks were found on any other islands surveyed at that time, including Gibbs Cay, Long Cay, and Salt Cay. Subsequent surveys by Reynolds et al. (2024a) on East Cay, Long Cay, Gibbs Cay (2022–2024), and Grand Turk (2024) failed to locate skinks anywhere other than Cotton Cay, where nine individuals were found on 4–5 March 2024. Reynolds et al. (2024a) concluded that Cotton Cay likely harbors the only remaining robust wild population of this species.

##### Habitat.

*Spondylurus turksae* has been found exclusively under or near rocks in open areas bordered by thick coastal scrub vegetation. Cotton Cay supports dense mixed graminoid and evergreen coastal scrub with abundant surface limestone rocks. The presence of rock-wall ruins provides substantial surface cover, which probably benefits the skink population there.

##### Natural history.

Very little is known about the natural history of *Spondylurus turksae*. Field observations indicate that the species is active diurnally; Reynolds et al. (2024a) found active individuals during afternoon diurnal searches (1610–1840 h) and inactive under rocks during nocturnal surveys (2122–2132 h). Based on the biology of confamilial species, *S. turksae* is probably a viviparous forager of small arthropods. The first published photographs of live individuals of this species were provided in Reynolds et al. (2024a).

##### Conservation.

*Spondylurus turksae* is among the most critically endangered reptiles in the Caribbean. Based on surveys conducted between 2008 and 2024, the species appears to be restricted to a single known population on the privately-owned Cotton Cay, where rats (*Rattus rattus*) are abundant. Reynolds et al. (2024a) recommended that removal of rats and any remaining goats on Cotton Cay should be the top conservation priority for this species, followed by establishing additional populations on islands free from invasive mammals and with minimal human impact. Additional surveys of the Turks Cays are needed to determine whether any other populations remain, although given the absence of records from those islands during visits by experienced herpetologists between 2008 and 2024, undiscovered populations seem unlikely. Grand Turk and Salt Cay are heavily modified and are unlikely to retain robust skink populations given their long histories of intensive land use (Reynolds et al. 2024a). Establishing a formal species conservation action plan should be a priority for this species.

### Family Sphaerodactylidae—Dwarf geckos and relatives

#### Aristelliger hechti

Taxon classificationAnimaliaSquamataSphaerodactylidae

Schwartz & Crombie, 1975

8DD0F45A-914F-59E9-8370-F4D0E3274160

Fig. 7

##### Common name.

Caicos Croaking Gecko.

**Figure 7.**
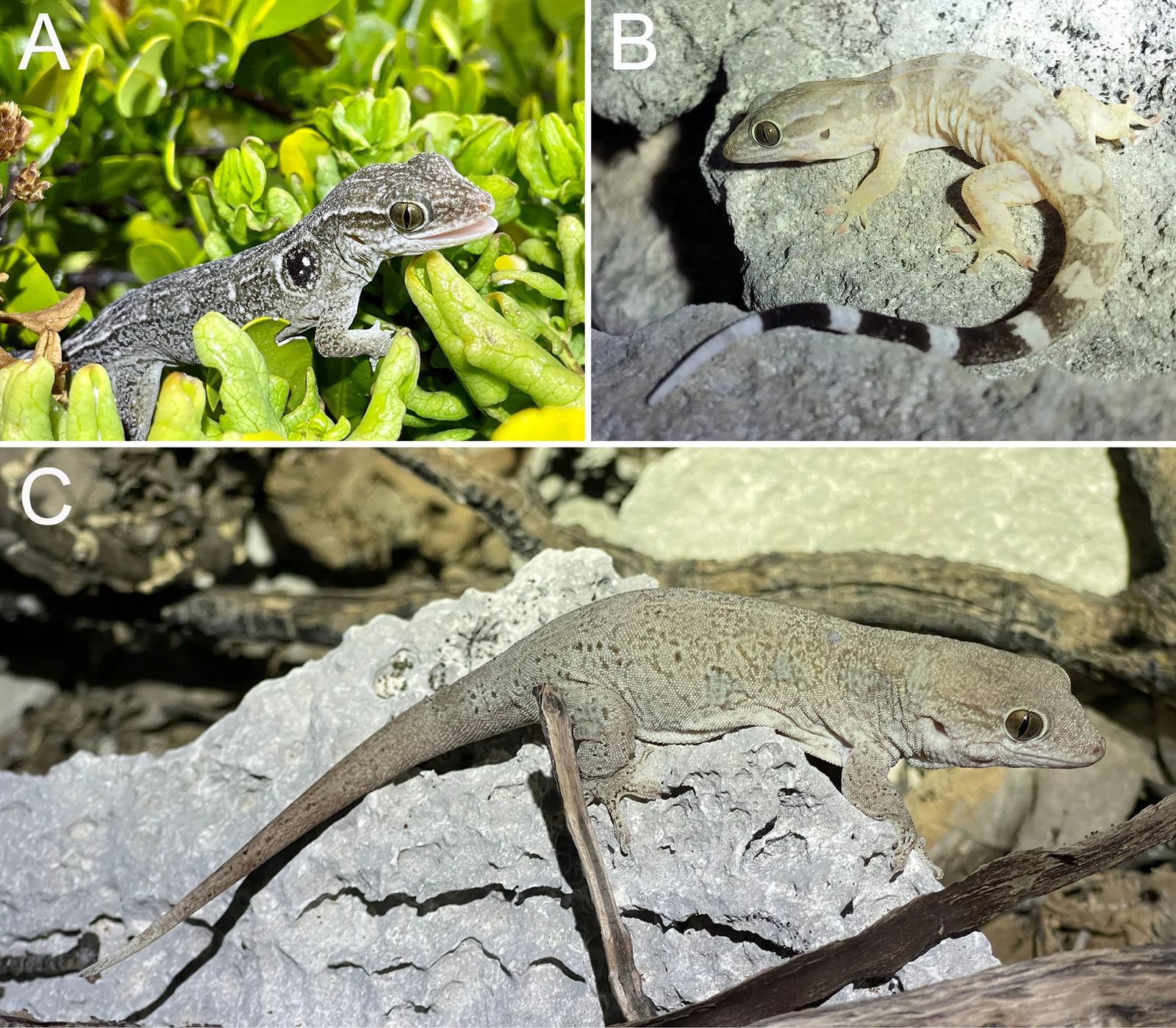
*Aristelliger hechti* from the Caicos Bank. **A, C**. Individuals from Big Ambergris Cay; **B**. Individual from North Caicos. Note the variation in dorsal coloration and the presence/absence of a scapular spot and striped tail.

##### Type material.

Holotype USNM 195844, Little Ambergris Cay.

##### IUCN status.

This species is considered Vulnerable (VU) on the IUCN Red List (Reynolds and Hedges 2016c).

##### Taxonomy.

Cochran (1934) first recorded *Aristelliger* (as *A. praesignis*) based on three specimens from Six Hills Cay off South Caicos. Hecht (1951) recognized the Caicos Island population as an undescribed species but did not assign a name or formal description; a task which was completed by Schwartz and Crombie (1975) based on additional specimens from Six Hills Cay. *Aristelliger hechti* is most closely allied to *A. lar* of Hispaniola (Schwartz and Crombie 1975; Bauer and Russell 1993).

##### Endemic status.

Endemic to the Caicos Bank.

##### Description.

*Aristelliger hechti* is a medium-sized gecko with variable coloration that is typically gray to brownish-tan or olive on the dorsum with variable speckling and banding. Large dark scapular ocelli often surrounded by a pale ring and including a whitish central spot are reduced or absent in some individuals (pers. obs.; Fig. 7). The head and body usually have middorsal bands or a series of fused pale rhomboids, but some individuals are patternless (Fig. 7; Schwartz and Crombie 1975; Bauer and Russell 1993). The tail can be plain or with light and dark bands (Fig. 7).

Males reach 90 mm SVL (64–90 mm; mean 80.7 mm) and females reach 75 mm SVL (57–75 mm; mean 66.8 mm) (Rabb and Hayden Jr 1957; Schwartz and Crombie 1975; Schwartz and Henderson 1991; Bauer and Russell 1993). Head lengths are 17–23 mm in males (mean 20.4 mm) and 15–19 mm in females (mean 17.4 mm). Head widths are 13–18 mm in males (mean 15.3 mm) and 11–15 mm in females (mean 12.8 mm). Adhesive toe pads are well-developed but lack terminal sheath-like structures (“friction pads”) on most digits (Hecht 1952; Schwartz and Crombie 1975). This species possesses the fragile skin characteristic of the genus *Aristelliger*, which tears easily when the animal is handled (Bauer and Russell 1992).

##### Distribution in the TCI.

*Aristelliger hechti* is restricted to the Caicos Bank (Reynolds 2011). The species is known from Big Ambergris Cay (where it is common), Little Ambergris Cay, French Cay, Six Hill Cays, East Caicos, and North Caicos (Schwartz and Crombie 1975; Bauer and Russell 1993; Reynolds 2011). Populations possibly occur on Middle Caicos, although this species has not been confirmed during herpetological surveys there. Occurrences on additional Caicos Bank islands remain uncertain. Burns et al. (1992) and Powell and Parmerlee Jr (1992) suggested that *A. hechti* might have been introduced to Cayos Siete Hermanos, Dominican Republic, although this has not been definitively confirmed (Schwartz and Crombie 1975; Bauer and Russell 1993).

##### Habitat.

*Aristelliger hechti* is most commonly found in xeric scrub on smaller islands such as French Cay and Big Ambergris Cay and in tropical dry forest on North Caicos. The species occupies vertical surfaces, including rock faces, trunks and branches of trees, rock walls, and the exterior of buildings. Diurnal refugia include rock piles, limestone crevices, and the interiors of hollow vegetation or wood. Nocturnal foraging occurs on vegetation, rock walls, and surface rocks, but also on artificial cover objects such as boards, corrugated metal roofing, and building exteriors.

##### Natural history.

The biology of *A. hechti* is poorly understood compared to many other species of Caribbean geckos (Reynolds 2011). Based on field observations and related congeners, the species is considered a nocturnal insectivore that feeds on a variety of arthropods. Bauer and Russell (1992, 1993) discussed the adaptive significance of skin fragility as a defensive mechanism that allows the animal to escape from predators, a mechanism analogous to caudal autotomy. The reproductive biology and population ecology of this species in the TCI are unknown.

##### Conservation.

*Aristelliger hechti* is likely a conservation concern due to its restricted range (confined to the Caicos Bank), apparently low population densities, and the presence of feral mammals on some islands within its range. An additional concern comes from the widespread introduction of the gecko *Hemidactylus mabouia* to the TCI, which is ecologically similar to *A. hechti* (Reynolds and Niemiller 2010). Additional research on the distribution, ecology, and reproductive biology of this species is needed.

#### Sphaerodactylus caicosensis

Taxon classificationAnimaliaSquamataSphaerodactylidae

Cochran, 1934

3B8ED7CF-82AB-5B01-A323-B0A3BC8E7B43

Fig. 8

##### Common name.

Caicos Geckolet.

**Figure 8.**
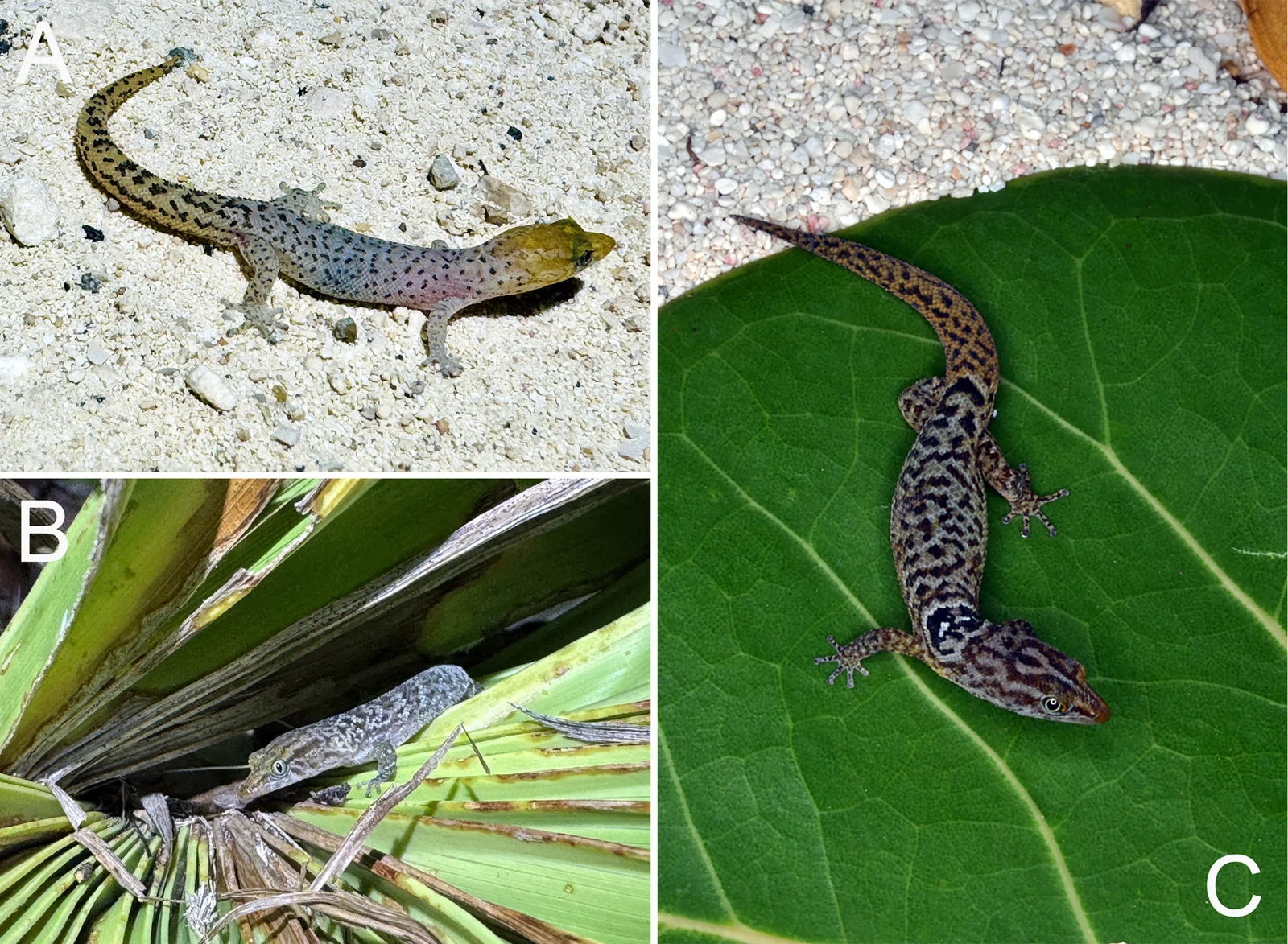
*Sphaerodactylus caicosensis* from the Caicos Bank. **A**. Adult male; **B, C**. Adult females. All individuals from Big Ambergris Cay.

##### Type material.

Holotype USNM 81443, South Caicos.

##### IUCN status.

This species is assessed as Least Concern (LC) on the IUCN Red List (Reynolds 2016b).

##### Taxonomy.

Cochran (1934) considered the species to be similar to *S. argus* (Cuba and Jamaica), as well as *S. corticola* and *S. mariguanae* (Bahamas), although its phylogenetic placement within this group is uncertain (Schwartz 1968).

##### Endemic status.

Endemic to the Caicos Bank.

##### Description.

*Sphaerodactylus caicosensis* is a very small lizard with a maximum SVL of ~32 mm. The dorsal coloration is variable but typically is a grayish ground color with dark spots or bands. Males typically have very small black spots resembling freckles, a dull yellow head, and a dull yellow tail. Females have a more pronounced pattern on the head and dorsum, occasionally bearing scapular spots.

##### Distribution in the TCI.

This species is restricted to the Caicos Bank and occurs on all major islands and some small cays and rocky islands with some vegetation (Reynolds 2011). The species is recorded from North Caicos, Middle Caicos, Providenciales, South Caicos, East Caicos, and West Caicos, as well as on the Caicos Cays (including Pine Cay and Little Water Cay), Big and Little Ambergris Cays, and Long Cay (Buckner et al. 2012).

##### Habitat.

*Sphaerodactylus caicosensis* is abundant in a wide variety of habitats, including xeric scrub, tropical dry forest, beaches, and areas with human habitation. Individuals are frequently found under rocks and litter along beaches and roads, and are commonly encountered on rock walls, low vegetation, and on stones at night. Many populations are edificarian and thrive near human habitation, where they use the crevices of buildings, rock walls, and accumulated debris as refugia. The species is tolerant of areas with high densities of feral mammals.

##### Natural history.

Like other congeners, *S. caicosensis* feeds on small arthropods and can be found active during the day under leaf litter or at night on the surface. Reproductive biology likely follows the sphaerodactylid pattern of producing single-egg clutches multiple times per year, with eggs deposited in moist soil, under bark, or in accumulated debris (Henderson and Powell 2009).

##### Conservation.

*Sphaerodactylus caicosensis* does not appear to be a conservation concern at present. The species is common across a wide range of habitats on the Caicos Bank and appears tolerant of disturbed habitats and human presence (Reynolds 2011).

#### Sphaerodactylus underwoodi

Taxon classificationAnimaliaSquamataSphaerodactylidae

Schwartz, 1968

6E5C01FD-2760-5232-81EF-1A381B9D017C

Fig. 9

##### Common name.

Turks Island Geckolet.

**Figure 9.**
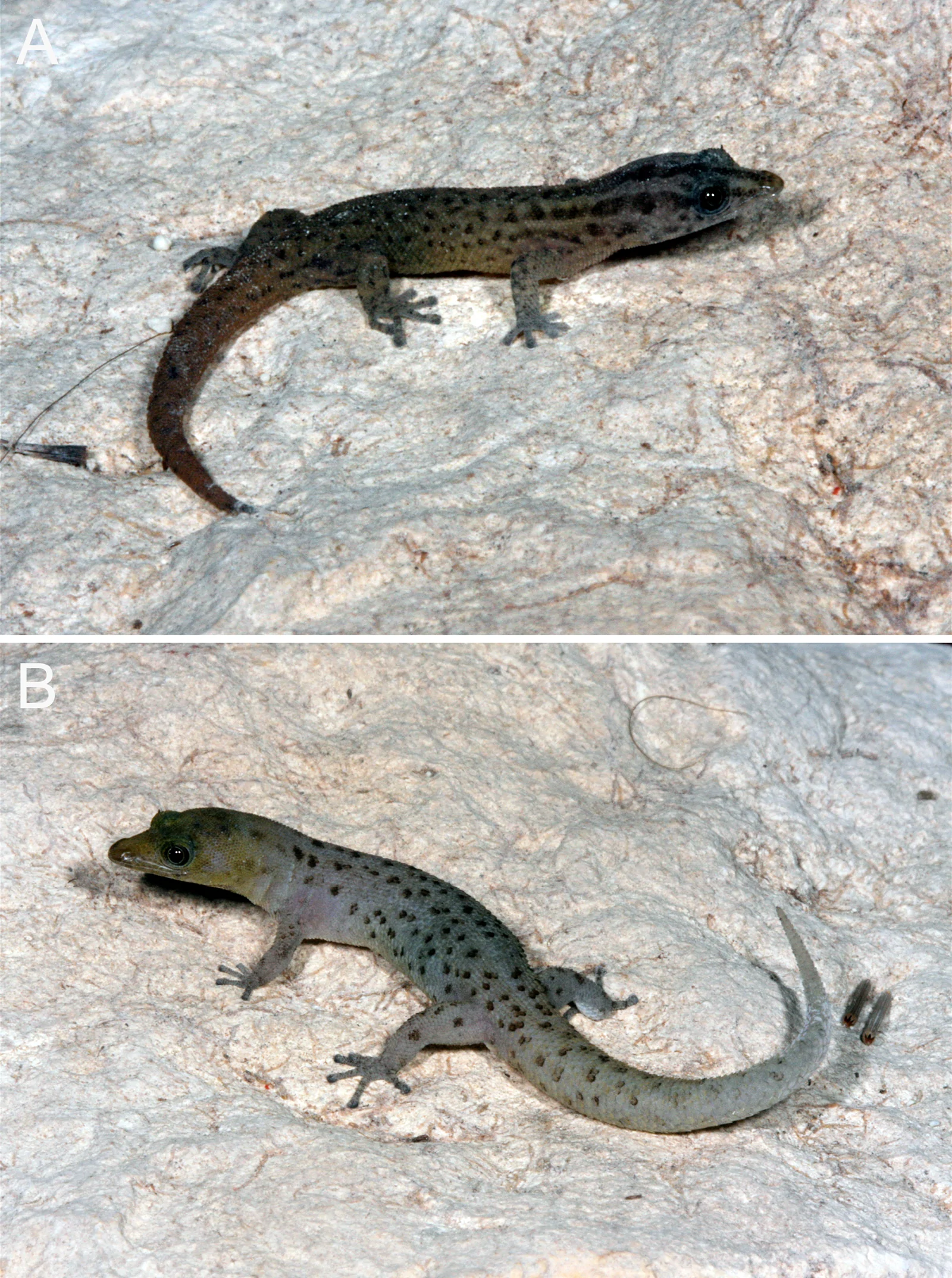
*Sphaerodactylus underwoodi* from Salt Cay. **A**. Adult female; **B**. Adult male. Photographs by Matthew L. Niemiller.

##### Type material.

Holotype CM 40637, Grand Turk.

##### IUCN status.

This species is assessed as Least Concern (LC) on the IUCN Red List (Reynolds 2016c).

##### Taxonomy.

This species is possibly most closely related to *S. difficilis* of Hispaniola and *S. inaguae* (Bahamas), rather than to *S. caicosensis* (Schwartz 1968), although phylogenetic studies using molecular data have not been published.

##### Endemic status.

Endemic to the Turks Bank.

##### Description.

*Sphaerodactylus underwoodi* has a maximum SVL of ~30 mm in males and 28 mm in females (Schwartz 1968). The species has a gray-to-brownish dorsal ground color with variable mottling and spotting. Males tend to have smaller spots and reduced patterning, giving them a salt-and-pepper appearance, along with a dull yellow color on the head and/or tail (Schwartz 1968; Schwartz and Henderson 1991). Females have more elaborate dorsal patterns, producing a mottled appearance with dark stripes on the sides of the head.

##### Distribution in the TCI.

*Sphaerodactylus underwoodi* has been recorded from Grand Turk, Salt Cay, Gibbs Cay, Long Cay, East Cay, and the Big Sand Cay group (Reynolds 2011, 2023; Buckner et al. 2012).

##### Habitat.

*Sphaerodactylus underwoodi* is edificarian, xerophilic, and is commonly found beneath rocks and litter in areas behind beaches and in urban lots with some vegetation, but also in crevices of walls, buildings, and rock outcrops. On East Cay, *S. underwoodi* was abundant underneath limestone rocks on hillsides away from ocean spray zones (Reynolds 2023), and on Salt Cay, the species was commonly found under rocks behind the wrack lines of beaches (Reynolds 2011).

##### Natural history.

The natural history of *S. underwoodi* is undocumented. Like other congeners, the species is a small insectivore, presumably feeding on arthropods found in leaf litter and rock crevices (Henderson and Powell 2009). Reproductive biology is expected to follow the multiple-clutch, single-egg clutch pattern characteristic of the family.

##### Conservation.

*Sphaerodactylus underwoodi* does not appear to be a conservation concern at present. The species is relatively common across the Turks Bank, tolerant of disturbed habitats and human presence, and found on heavily developed Grand Turk.

### Family Typhlopidae—Blindsnakes

#### Antillotyphlops platycephalus

Taxon classificationAnimaliaSquamataTyphlopidae

(Duméril & Bibron, 1844)

F8DB34C4-4249-51BC-8458-C0DA0F9D87CF

Fig. 10

##### Common name.

Puerto Rican White-tailed Blindsnake; Caicos Blindsnake.

**Figure 10.**
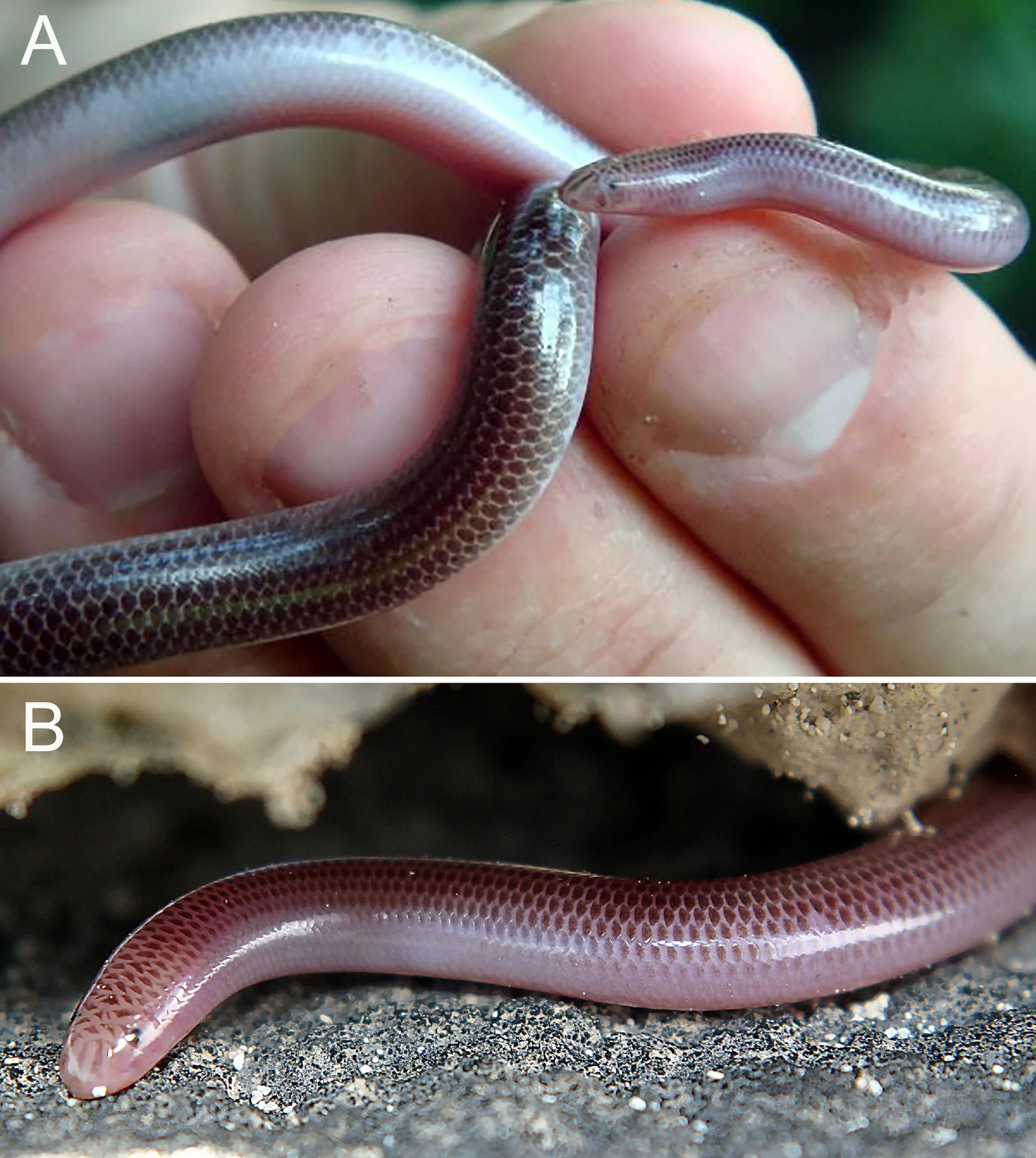
*Antillotyphlops platycephalus* from the Caicos Bank. **A**. An individual from North Caicos; **B**. An individual from Big Ambergris Cay. The top image was slightly enhanced in Adobe Photoshop to remove distracting foreground material.

Photographic voucher APSU 18948 (Big Ambergris Cay).

##### IUCN status.

This species has been evaluated as Least Concern (LC) on the IUCN Red List (Reynolds 2019).

##### Taxonomy.

The blindsnake populations of the TCI were formerly referred to as *Typhlops richardii* in Schwartz and Henderson (1991) and as *T. platycephalus* by Hedges and Thomas (1991). Reynolds (2011) suggested that the TCI populations might be assigned to a unique species based on DNA sequence data and considered them *Typhlops cf. platycephalus*. However, subsequent molecular genetic work (RGR, unpubl.; cytochrome *b* locus GenBank #PZ353239) has not been conclusive in this regard. The generic name was changed to *Antillotyphlops* by Hedges et al. (2014), which includes 11 other West Indian species (Uetz et al. 2026).

Both Hedges et al. (2014) and Nagy et al. (2015) included the species in a large-scale molecular phylogeny of blindsnakes, showing it as a sister to *A. naugus* from the British Virgin Islands, although they used specimens from Puerto Rico and not the TCI (SBH 172180 and USNM FS172194, respectively).

##### Endemic status.

The species is not endemic to the TCI and is also found in Puerto Rico.

##### Description.

The Caicos Blindsnake is a small fossorial snake with reduced eyes visible as dark spots under the scales, smooth scales, and a relatively blunt, flat head (*platycephalic* means “flat-headed”), and a tail of similar proportions (Fig. 10). Dorsal and ventral coloration is generally dark rose-to-pinkish in the Caicos Islands, although red-brown or dark-brown in Puerto Rico (Hedges et al. 2014). The tail does not appear to have the strongly white-banded pattern seen in Puerto Rican populations. The rostral scale is large and projects slightly forward, facilitating burrowing. The maximum adult size is poorly documented for the TCI population, but the species in Puerto Rico reaches a total length of 302 mm, whereas that of congeners ranges from 110 to 360 mm (Hedges et al. 2014). One individual from Big Ambergris Cay (Oct. 2015) measured 305 mm SVL, 7 mm TL, and 8.5 g (pers. obs.).

##### Distribution in the TCI.

In the TCI, the Caicos Blindsnake is restricted to the Caicos Bank (Reynolds 2011). Although Hedges et al. (2014) listed the species as found on North Caicos and Providenciales, it has never been recorded from the Turks Bank. It is known from North Caicos (where individuals were found in tropical dry forest) and Big Ambergris Cay, where individuals have been found underneath rocks in sandy soil in low scrub as well as crawling on the surface of limestone karst during heavy rain at night (Reynolds 2010). This fossorial and cryptic species likely occurs on additional islands of the Caicos Bank.

##### Habitat.

*Antillotyphlops platycephalus* has not been studied in the Caicos Islands, but it appears to be xerophilic and is fossorial, found underneath rocks in sandy soil in low scrub and in tropical dry forest on North Caicos, Providenciales, and Big Ambergris Cay. Individuals are occasionally found on the surface at night on Big Ambergris Cay.

##### Natural history.

Little is specifically known about the natural history of this species in the TCI. Like other typhlopids, the Caicos Blindsnake is assumed to be a fossorial insectivore feeding on social insect larvae (ants and termites) in underground galleries (Henderson and Powell 2009). The species is oviparous. Caicos Blindsnakes are generally uncommon or rarely encountered in the field (pers. obs.), although this could reflect their cryptic fossorial lifestyle rather than genuine rarity.

##### Conservation.

The Caicos Blindsnake does not appear to be of conservation concern at the range-wide level. Nevertheless, the species is likely rare in the TCI, and habitats where it has been regularly found on Big Ambergris Cay are subjected to heavy development. Whether this species would suffer from competition or displacement with the introduced Brahminy Blind Snake (*Indotyphlops braminus*), which is established and common on Grand Turk and Providenciales (Reynolds and Niemiller 2010; Reynolds 2011), is unknown.

### Family Tropidophiidae—Tropes

#### Tropidophis greenwayi greenwayi

Taxon classificationAnimaliaSquamataTropidophiidae

Barbour & Shreve, 1936

F78A1FB4-C0C1-5D3D-AF59-8A3E3FB17279

Fig. 11B–D

T. g. greenwayi Holotype MCZ 42051, Big Ambergris Cay.

##### Common name.

Caicos Trope.

**Figure 11.**
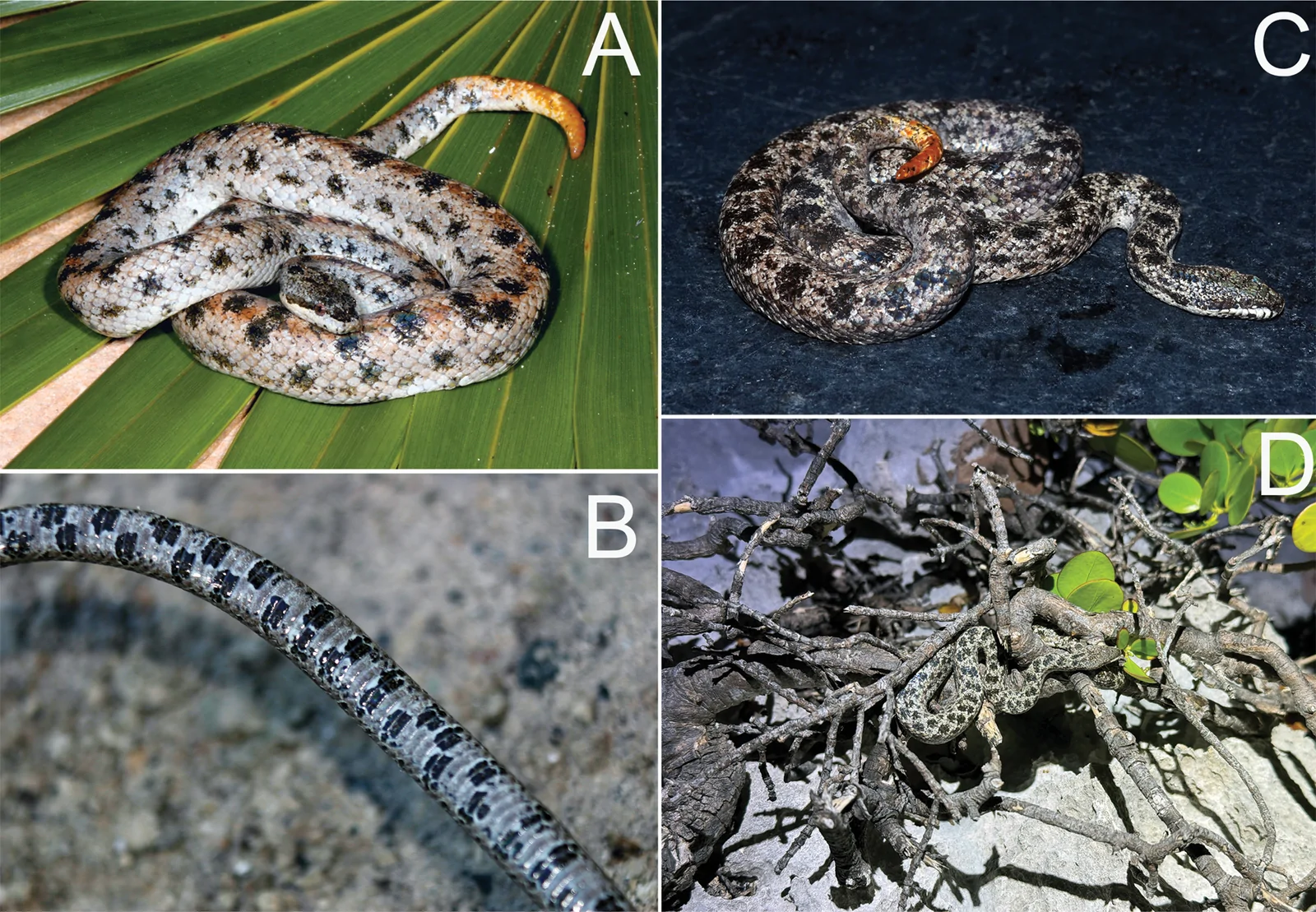
*Tropidophis greenwayi* from the Caicos Bank. **A**. *T. g. lanthanus* from North Caicos. Photograph by Matthew L. Niemiller; **B**. Underside of *T. g. greenwayi* from Big Ambergris Cay showing ventral pattern; **C**. *T. g. greenwayi* from Big Ambergris Cay; **D**. *In situ* individual from Big Ambergris Cay.

#### Tropidophis greenwayi lanthanus

Taxon classificationAnimaliaSquamataTropidophiidae

Schwartz, 1963

F7F374C4-C189-57C9-A217-BC5E95E7E3DC

Fig. 11A

T. g. lanthanus Holotype MCZ 69630, South Caicos.

##### Common name.

Caicos Trope.

##### IUCN status.

This species has been assessed as Vulnerable (VU) on the IUCN Red List (Reynolds 2021).

##### Taxonomy.

*Tropidophis greenwayi* is endemic to the Caicos Bank and includes two currently recognized subspecies: *T. g. greenwayi* Barbour & Shreve, 1936 (type locality Big Ambergris Cay; MCZ 42051) and *T. g. lanthanus* Schwartz, 1963 (type locality South Caicos; MCZ 69630). The former subspecies tends to be darker in overall coloration than the latter (Fig. 11), and Schwartz (1963) considered *T. g. lanthanus* to have a “dorsal pale zone” versus being completely mottled in *T. g. greenwayi*. This results in a series of more apparent dorsal blotches in *T. g. lanthanus* versus *T. g. greenwayi*, where the blotches are obscured by mottling (this is readily visible in Fig. 11). Schwartz also distinguished *T. g. greenwayi* as having a “salt-and-pepper” coloration on the head versus a more uniform brown in *T. g. lanthanus* (also somewhat apparent in Fig. 11). More tentatively, Schwartz considered the subspecies *T. g. lanthanus* to have a higher number of ventral scales (157–165) versus *T. g. greenwayi*, although he merely “suspected” this to be the case owing to so few specimens of *T. g. greenwayi* being available (*n* = 2). Whether the subspecific designations are supported by molecular data remains to be determined.

##### Endemic status.

The species *Tropidophis greenwayi* is endemic to the Caicos Bank, and the subspecies *T. g. greenwayi* is endemic to Big Ambergris Cay.

##### Description.

*Tropidophis greenwayi* is a small, stout snake. The subcaudal scales are divided, and dorsal coloration is generally brown to grayish-brown with variable darker banding or blotching. The eyes are large with vertically elliptical pupils. Combined across both subspecies, adults range from 138–355 mm SVL and 155–410 mm TOL, with females significantly larger than males (Iverson 1986; Reynolds unpubl. data). Juveniles are 110–138 mm SVL and 125–155 mm TOL, and two near-term embryos measured 57.0 and 68.5 mm SVL (Iverson 1986). *Tropidophis g. greenwayi* from Big Ambergris Cay (*n* = 6) range from 279–288 mm SVL with masses ranging from 10.5–19.5 g (Reynolds, unpubl. data).

##### Distribution in the TCI.

*Tropidophis g. greenwayi* is endemic to Big Ambergris Cay, where only two specimens collected as types by Barbour and Shreve in 1936 were known despite searches in January of 1961 by Schwartz (1963) until an additional individual was found on 20 March 2009 (Reynolds et al. 2010). Six individuals have been observed between 2010 and 2025 (pers. obs.).

*Tropidophis greenwayi lanthanus* is found on the largest islands of the Caicos Bank, including Providenciales, North Caicos, Middle Caicos, South Caicos, and possibly East Caicos and West Caicos. It also has been recorded from Long Cay (Caicos Bank) and Middleton Cay (Iverson 1986; Reynolds 2011).

##### Habitat.

*Tropidophis greenwayi lanthanus* is relatively mesophilic, frequently occurring near sources of standing water or groundwater such as old wells, ephemeral ponds, and thick forest on North and Middle Caicos (Reynolds 2011). Adults are particularly abundant inside the walls of old stone wells where metamorphosing Cuban Treefrogs (*Osteopilus septentrionalis*) emerge from the water below (Manco 2006; Reynolds 2011). Individuals are occasionally found in more xeric situations, such as rocky well-drained hillsides on Providenciales and Middle Caicos (Reynolds 2011) and open xeric shrubland on South Caicos (Iverson 1986). The subspecies *T. g. greenwayi* on Big Ambergris Cay is considered xerophilic, as mesophilic habitat is essentially absent on that island.

##### Natural history.

*Tropidophis greenwayi* is a nocturnal constrictor that feeds primarily on small vertebrates, particularly frogs and lizards. They feed on the Cuban Treefrog (*Osteopilus septentrionalis*), an introduced species that has become abundant in wells and cisterns on North and Middle Caicos (Tolson and Henderson 1993; Manco 2006; Reynolds and Niemiller 2010). Other prey items include *Anolis scriptus* (pers. obs.) and likely other small lizards. Like other species in the genus, this species exhibits autohemorrhagy (self-induced bleeding from the mouth, eyes, or cloaca), cloacal discharge, and defensive behaviors. Females give birth to live young.

##### Conservation.

*Tropidophis greenwayi* was at one time collected for the international pet trade, a practice that can occasionally devastate small island populations (Webb et al. 2002). A former TCI government official reportedly granted reptile dealers a permit to remove thousands of individuals from North Caicos, with the apparent suggestion that removing all the snakes would be acceptable (Reynolds 2011). The species is likely susceptible to introduced mammalian predators, and the Long Cay population might be benefiting from the removal of cats from that island (Mitchell et al. 2002). The subspecies *T. g. greenwayi* is restricted to Big Ambergris Cay, which, while currently free of invasive predators, is currently undergoing heavy tourist development (Reynolds et al. 2024b).

### Family Boidae—New World boas

#### Chilabothrus chrysogaster chrysogaster

Taxon classificationAnimaliaSquamataBoidae

(Cope, 1871)

B04BD484-586C-52D1-867D-A8EE2EDF4162

Fig. 12

##### Common name.

Turks and Caicos Boa.

**Figure 12.**
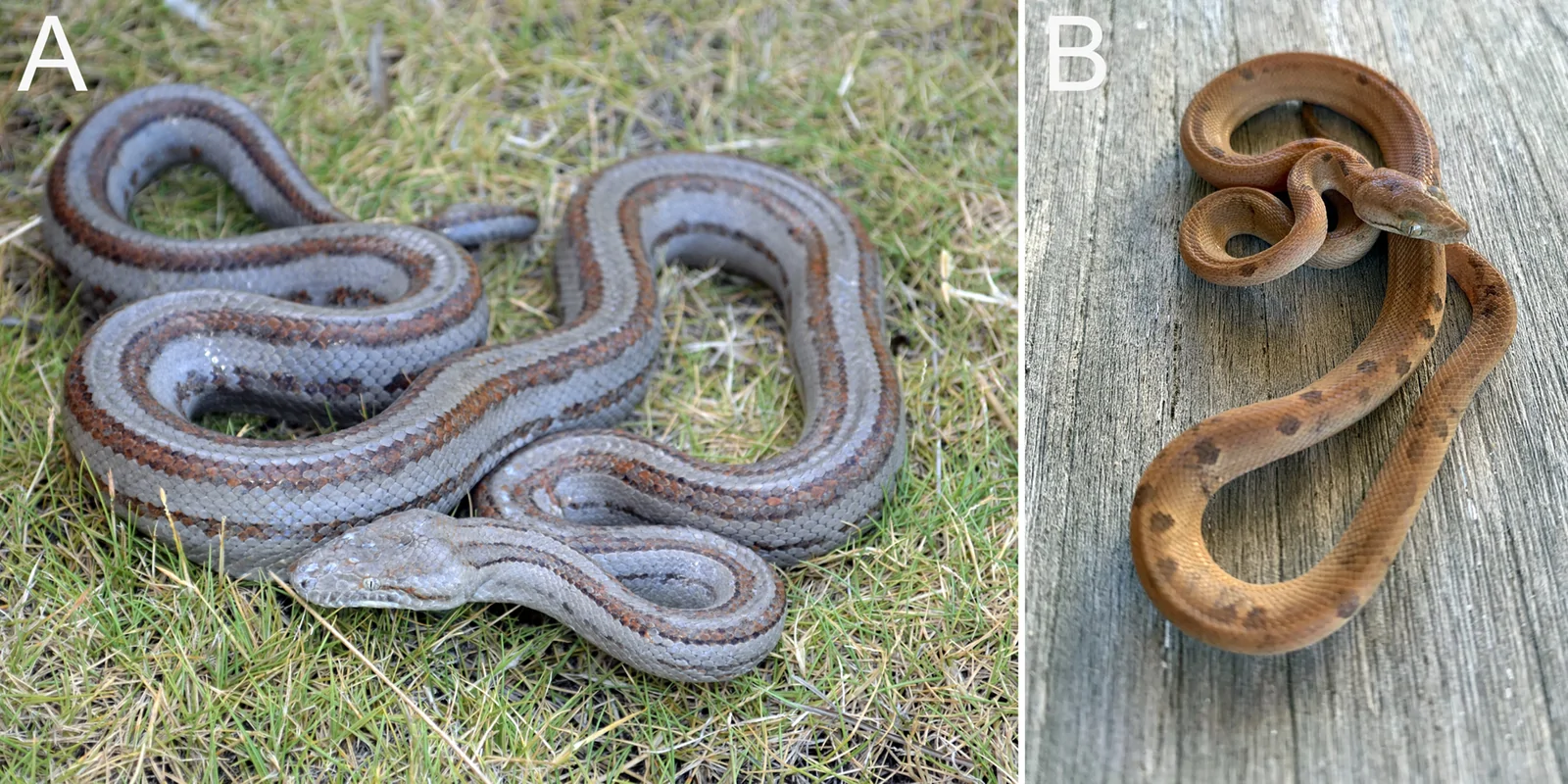
*Chilabothrus c. chrysogaster* from Big Ambergris Cay, Caicos Bank. **A**. Adult female with a brown striped pattern and gray background coloration; **B**. Yearling from Big Ambergris Cay, Caicos Bank, showing juvenile orange coloration and spotted pattern.

##### Type material.

Holotype ANSP 10322, “Turks Island”.

##### IUCN status.

This species has been assessed as Near Threatened (NT) on the IUCN Red List (Reynolds and Buckner 2021).

##### Taxonomy.

Two subspecies are recognized, *C. c. chrysogaster* from the TCI and *C. c. relicquus* from Great Inagua, Bahamas (Reynolds et al. 2023). The species is derived from an independent colonization event from Hispaniola and is sister to the Bahamian/Hispaniolan congeners (Reynolds et al. 2013).

##### Endemic status.

*Chilabothrus c. chrysogaster* is endemic to the TCI.

##### Description.

*Chilabothrus chrysogaster* is sexually dimorphic, with males reaching less than 850 mm SVL and females over 1300 mm SVL. Mean adult female SVL is 721 mm with a mass of 123 g and mean male SVL is 652 mm with a mass of 70 g (Reynolds et al. 2023). Neonates are born at ~ 280–300 mm SVL and 6–8 g in mass (Reynolds and Deal 2010). Males frequently have slightly visible cloacal spurs.

Coloration in *C. c. chrysogaster* is highly variable (Reynolds et al. 2020b, 2023). The venter is cream-colored with variable amounts of gray to brown freckling. The dorsal background color ranges from light gray to tan with a series of darker blotches or stripes. Striped morphs (~ 15% of the Big Ambergris population) have two or four dark lines, each four scales wide, running longitudinally from the base of the head along the dorsum and sides to the tip of the tail. Spotted morphs exhibit highly variable blotch sizes, shapes, and distribution. Dark postocular stripes one scale wide extend from the back of the orbit to the jaw, tapering to a fine point over the quadrate bone. Neonates are born with an orange ground color on both the venter and sides, with cream-colored venters lacking stippling. Dorsal color transitions from orange to gray, and dorsal pattern colors darken toward the end of the first year, with color change complete once a size of ~ 500 mm SVL is reached, probably at ~ 1–2 years of age (Reynolds et al. 2023).

##### Distribution in the TCI.

*Chilabothrus chrysogaster* occurs on both the Turks and Caicos banks. The Grand Turk population is considered extirpated (Reynolds 2011, 2023), with the species not recorded there despite focused surveys (Reynolds and Henderson 2026), and an anecdotal report in 2021 could have represented an introduced cornsnake (*Pantherophis guttatus*). The species is known to occur on East Cay and Gibbs Cay in the Turks Islands (Reynolds 2023). On the Caicos Bank, it has been recorded from nine islands, including Providenciales, North Caicos, Middle Caicos, and East Caicos. However, boas are considered rare on Providenciales, given the scale of human development. Smaller islands with populations include Dellis Cay and Parrot Cay (Caicos Cays), Joe Grant’s Cay (near East Caicos), and the Ambergris Cays (Reynolds and Gerber 2012).

##### Habitat.

*Chilabothrus c. chrysogaster* occupies habitats ranging from dense, relatively humid closed canopy blackland coppice forest on North Caicos to xeric scrub vegetation on Big Ambergris Cay and rock outcrops on East Cay (Reynolds et al. 2023). Boas occasionally are found foraging on dunes, in red mangroves (*Rhizophora mangle*), or on rocky ironshore areas very close to the sea (Reynolds et al. 2024b). On small islands such as Gibbs Cay, boas are found foraging at the bases of cacti or in coastal scrub (Reynolds et al. 2023). Large females often rest during the day partially under fallen leaves at the base of thick bushes, where they are nearly invisible (Reynolds et al. 2024b). Boas occasionally occur in sandy *Coccothrinax*-dominated forests, although at much lower densities than in coppice forests (Reynolds and Gerber 2012).

##### Natural history.

Between 2007 and 2025, ongoing surveys marked over 2,500 boas, yielding an estimate of 9,935 ± 740 boas on Big Ambergris Cay, a density of ~ 25 boas/ha (Reynolds and Henderson 2026). A maximum of 52 boas were found in three hours of nocturnal searching on one night in August (Reynolds et al. 2023). On the larger Caicos Bank islands, boas are generally uncommon, although some local populations of higher density are known from coppice forest. On Providenciales, population densities are very low in some areas. On Gibbs Cay, no more than three boas have been found during a single 24-hour survey, but two or three are usually found per survey visit (Reynolds 2023). The East Cay population on Turks Bank is thought to be quite dense based on encounter rates, but it has been surveyed only twice (Reynolds 2023).

A 20-month radiotelemetry study of 19 adult female *C. c. chrysogaster* on Big Ambergris Cay (Reynolds et al. 2024b) showed mean female home ranges of 1.83 ha (range 0.88–3.99 ha), with core space use averaging 0.39 ha. Female home ranges overlap substantially in both time and space. Radio-tracked boas were observed foraging in bushes and shrubs, on the ground in open rocky areas, in coppice forests, in red mangroves, and in coastal wrack (Reynolds et al. 2024b). This species is mainly saurophagous, with a diet strongly structured by ontogeny and sex. Individuals slowly search rock crevices for sleeping lizards, frequently observed stalking active prey such as *Aristelliger hechti* on rock walls, although several instances of carrion consumption have been documented (Reynolds et al. 2023). Large females are big enough to consume juvenile *Cyclura carinata* iguanas (to ~1.5 kg).

Breeding occurs in spring, and boas are more active from March through August. Young born in the fall reach ~ 350 mm SVL and 9–16 g by March, at which time they begin to lose juvenile coloration (Reynolds et al. 2023). An estimated growth rate of ~ 20.4 mm SVL per month was calculated from newborns and young-of-year snakes from North Caicos (Reynolds and Deal 2010), consistent with earlier estimates (Buden 1975). Neonates are arboreal and likely feed on young *Anolis* lizards, *Sphaerodactylus*, and small *Aristelliger* geckos. Yearlings frequently occupy large rock piles in great numbers, where they feed on abundant *Sphaerodactylus*, *Aristelliger*, and introduced *Hemidactylus* geckos (Reynolds et al. 2023).

##### Conservation.

The largest threats to this subspecies are predation by feral cats and habitat modification for development. The absence of boas from islands with high rat densities such as Cotton Cay is suggestive of rat predation, although the persistence of *S. turksae* on that island indicates the relationship is not straightforward and warrants direct investigation. Only the largest females could potentially subdue and consume full-grown rats (Reynolds et al. 2023). Snakes are routinely found dead on roads, including during periods of peak development on Big Ambergris Cay (2006–2008 and 2015–2025).

## Summary

The terrestrial reptilian fauna of the TCI includes 11 native species, of which seven are of some conservation concern (Fig. 2). These species of conservation concern reveal a pattern whereby populations that were historically distributed across both banks have been extirpated from islands with a long history of human disturbance and now persist primarily on smaller, more remote cays (Iverson 1978; Reynolds 2011). *Cyclura carinata* has been extirpated from nearly all large islands and now occupies less than 5–10% of its former range (Gerber et al. 2020). *Chilabothrus c. chrysogaster* has been extirpated from Grand Turk and South Caicos and is considered rare across much of Providenciales (Reynolds 2011; Reynolds et al. 2023). *Leiocephalus psammodromus* has been extirpated from four islands where it was previously recorded, and *Spondylurus turksae* appears to be restricted to a single remaining population on Cotton Cay (Reynolds et al. 2024a). The primary driver of these extirpations is well established and consistent across species and islands: the introduction of feral cats and rats, which prey on native reptiles and effectively prevent juvenile recruitment into populations (Iverson 1978; Mitchell et al. 2002; Reynolds 2011). Habitat loss from development and vehicle mortality represent additional threats (Reynolds 2011; Reynolds et al. 2023).

While not discussed here, at least twelve introduced reptilian species are now established or have been documented in the TCI, and their ecological interactions with native fauna remain unstudied (Reynolds and Niemiller 2010). The potential for competitive displacement of *Aristelliger hechti* by *Hemidactylus mabouia* and of the native blindsnake *Antillotyphlops platycephalus* by *Indotyphlops braminus* warrants further attention.

Long-term population monitoring programs, now ongoing for over 18 years for *C. c. chrysogaster* on Big Ambergris Cay (Reynolds et al. 2023) and for over 30 years for *C. carinata* (Gerber et al. 2020), are providing baseline data necessary to detect population trends and inform management decisions. Public awareness of the native herpetofauna has increased substantially in the last two decades through ecotourism, media campaigns, interpretive programs, and the designation of *C. carinata* as a natural symbol of the TCI (Reynolds 2011). The future of the native herpetofauna of the Turks and Caicos Islands will depend on continued enforcement of existing wildlife protection laws, expansion of feral mammal eradication programs to additional islands with suitable habitat, prevention of new introductions through improved biosecurity measures at ports of entry, and sustained research effort on the species for which basic natural history data remain limited.

## Supplementary Material

XML Treatment for Cyclura carinata

XML Treatment for Leiocephalus psammodromus

XML Treatment for Leiocephalus psammodromus psammodromus

XML Treatment for Leiocephalus psammodromus apocrinus

XML Treatment for Anolis scriptus scriptus

XML Treatment for Spondylurus caicosae

XML Treatment for Spondylurus turksae

XML Treatment for Aristelliger hechti

XML Treatment for Sphaerodactylus caicosensis

XML Treatment for Sphaerodactylus underwoodi

XML Treatment for Antillotyphlops platycephalus

XML Treatment for Tropidophis greenwayi greenwayi

XML Treatment for Tropidophis greenwayi lanthanus

XML Treatment for Chilabothrus chrysogaster chrysogaster

## Additional information

### Artificial Intelligence (AI) use

The author accepts full responsibility for the content of the manuscript, including the disclosure of any use of AI.

Regarding the use of AI in the preparation of this manuscript, the author declare the following: Claude. Used for: Copy editing of the text, literature cited checking, and preparation of fig. 2 from original data.

### Author ORCIDs

R.G. Reynolds https://orcid.org/0000-0002-0056-8304

### Data availability

All of the data that support the findings of this study are available in the references or from the author by request.
